# Machine Learning-Based Fluorescence Assessment for Augmented Imaging and Decision Support in Glioblastoma Resections

**DOI:** 10.3390/cancers18071125

**Published:** 2026-03-31

**Authors:** Anna Schaufler, Klaus-Peter Stein, Sunisha Pamnani, Claudia A. Dumitru, Belal Neyazi, Ali Rashidi, Axel Boese, I. Erol Sandalcioglu

**Affiliations:** 1 Department of Neurosurgery, Faculty of Medicine, Otto-von-Guericke University Magdeburg, Leipziger Strasse 44, 39120 Magdeburg, Germany; 2 INKA Health Tech Innovation Laboratory, Medical Faculty, Otto-von-Guericke University Magdeburg, Leipziger Strasse 44, 39120 Magdeburg, Germany

**Keywords:** glioblastoma surgery, fluorescence-guided surgery, maximally safe resection, machine learning, autoencoder

## Abstract

**Background/Objectives**: Glioblastoma is the most common and aggressive primary malignant brain tumor in adults, characterized by infiltrative growth and poor prognosis. Achieving maximal resection without inducing neurological deficits remains a challenge in glioblastoma surgery. While 5-aminolevulinic acid-based fluorescence-guided surgery supports intraoperative tumor visualization, its reliability is limited by patient variability and weak fluorescence signals. This study proposes a machine learning framework to enhance fluorescence-guided surgery sensitivity by analyzing surgical microscope images at the pixel level. **Methods**: Fluorescence-mode neurosurgical microscope images of synthetic samples with known Protoporphyrin IX (PPIX) concentrations were used to train three classifiers (Support Vector Machine, Naïve Bayes, Neural Network) for pixel-wise fluorescence detection. In parallel, three contrastive-learning-based Variational Autoencoders (VAE, β = 1, 2, 3) were evaluated for detecting weak fluorescence beyond visual perception. Additionally, a regression model was trained to relate pixel features to PPIX concentration. The best-performing VAE (β = 1) was subsequently trained on real intraoperative data, and its detection sensitivity was compared to annotations from four experienced surgeons. **Results**: The proposed model achieved the highest detection rates on synthetic test data when calibrated for 99% specificity. Applied to real intraoperative images, the model revealed fluorescent areas substantially larger than those marked by experienced surgeons. In non-5-ALA control cases, minimal false positives were observed, indicating a specificity exceeding 99.9%. The regression model reliably quantified PPIX concentration in synthetic samples ($R^{2}=0.92$). **Conclusions**: By enabling more sensitive and objective fluorescence detection, this approach offers a valuable tool for improving surgical decision-making and facilitating safer, more extensive tumor resections.

## Simple Summary

Glioblastoma is a highly aggressive brain tumor characterized by infiltrative growth, making complete surgical removal challenging without compromising critical brain functions. During surgery, 5-ALA fluorescence is commonly used to visualize tumor tissue; however, weak or ambiguous signals can limit its reliability. In this study, we developed a machine learning approach to enhance fluorescence detection in standard surgical microscope images. The proposed model analyzes images at the pixel level and is capable of identifying subtle fluorescence signals that may not be visible to the human eye. Experiments on synthetic data and real intraoperative images demonstrated that the method detects larger tumor-associated regions while maintaining very high specificity. In addition, a separate regression model was used to estimate PPIX-related fluorescence intensity in synthetic samples. Overall, this approach may support surgeons with more objective information, contributing to improved tumor resection while preserving healthy brain tissue.

## 1. Introduction

Despite ongoing efforts in basic and applied research, there is no curative treatment option for glioblastoma (GBM), the most common primary brain tumor in adults. The survival prognosis of patients at present remains as low as 5 to 15 months [1]. Due to its intratumoral heterogeneity and plasticity, combating the tumor with individual pharmacological agents has proven to be extremely difficult, and its stem cell-like characteristics further enable it to develop resistance to chemotherapy, radiation, and immunotherapy [2]. Although several prognostic factors influence the survival of patients with glioblastoma, including tumor location, MGMT promoter methylation, and age, the extent of surgical resection (EOR) is the only modifiable factor directly impacted by therapeutic intervention [3]. Maximally safe tumor resection has been consistently shown to improve patients’ quality of life (QoL), as well as prolong progression-free survival (PFS) and overall survival (OS) [4,5,6,7]. Furthermore, Grabowski et al. [8] and Gerritsen et al. [9] have presented the remaining tumor volume as an additional and potentially more meaningful prognostic factor for PFS and OS than EOR.

The assumption that remaining neoplastic cells in the brain are a major cause of glioblastoma relapse is supported by studies on supramarginal resection (SMR), in which cytoreduction is extended beyond tumor borders detected by MRI. Several studies comparing SMR with gross total resection (GTR)—defined as the removal of all visible tumor tissue—have reported significantly improved progression-free survival (PFS) and overall survival (OS) following SMR [7,10,11,12]. However, while maximizing the extent of resection is usually the primary goal of glioblastoma surgery, preventing iatrogenic neurological deficits resulting from radical tissue removal is equally crucial [9,13].

To enhance visual demarcation of tumor tissue for improved precision during resection, exogenous 5-aminolevulinic acid (5-ALA) is used as a surgical adjunct. 5-ALA is an approved fluorescent marker for high-grade gliomas that selectively labels neoplastic cells. After oral administration 3–4 h before surgery, it is metabolized within the heme biosynthesis pathway to the fluorescent molecule protoporphyrin IX (PPIX), which accumulates intracellularly in malignant glioma cells due to altered metabolism [14]. The use of 5-ALA in high-grade glioma is an established method, and contemporary neurosurgical microscopes are equipped with the necessary modalities for intraoperative fluorescence imaging [13,15].

PPIX fluorescence is excited by blue light around 405 nm and emits lower-energy light at approximately 635 nm, appearing pinkish red. Fluorescence-guided surgery (FGS) thus enables real-time visualization of the tumor independent of neuronavigation and brain shift [14].

Previous studies showed that tumor regions revealed by 5-ALA probably even exceed the preoperatively detected MRI contrast-enhanced volume [14,16]. Its role in achieving a greater EOR in malignant gliomas and the associated benefits in PFS and OS is strongly supported by numerous studies [17,18,19,20,21,22,23].

Despite these benefits, important limitations remain. Although the positive predictive value (PPV) of 5-ALA approaches 99%, the negative predictive value (NPV) ranges from 22% to 90%, indicating that tumor tissue may remain undetected [14]. Reported specificity and sensitivity range between 89 and 100% as well as 83 and 87%, respectively [24]. Clinically, most glioblastoma patients still develop recurrence near the resection cavity even after FGS [7,25]. Despite the use of fluorescent imaging, tumor cell residues were identified in more than 50% of biopsies taken from the tumor margin [16].

The fluorescence intensity in malignant glioma, and thus the ability to delineate tissue contaminated by tumor cells, is by no means homogeneous but is strongly related to tumor cell density and cell proliferation, among other factors [14,25]. In diffusely infiltrative tumor margins, fluorescence gradually fades, making tumor borders difficult to identify [25]. In addition, interpatient variability and procedural factors—including the timing of 5-ALA administration, surgical workflow variations, and microscope parameters such as illumination intensity, working distance, angle of incidence, shadowing, and photobleaching—further influence fluorescence visibility [14,24,26]. There is an apparent need for improved visualization of active tumor areas that exceeds the capabilities of basic 5-ALA fluorescence imaging in terms of sensitivity as well as objectivity [14].

Most research attempts to meet the need for improved FGS imaging involve spectroscopic solutions and can be grossly divided into hyperspectral wide-field camera technologies and spectroscopic probe (SP)-based approaches. A review article on probe-based spectroscopy of 5-ALA-induced fluorescence is available from Gautheron et al. [27]. The sensitivity of fluorescence detection with SPs substantially exceeds that of conventional FGS. SPs can detect fluorescence signals emanating from a tissue surface within the sub-millimeter range and additionally enable quantification of PPIX in the tissue, providing a more objective tissue assessment [28]. However, probe-based approaches are limited to localized measurements rather than a comprehensive scan of the resection cavity, significantly reducing their practical utility.

Hyperspectral imaging (HSI) enables wide-field capture, encompassing the entire surgical field similarly to a surgical microscope. In their 2022 study, Lehtonen et al. [29] were able to demonstrate that HSI has a considerably lower sensory threshold for PPIX fluorescence compared to visual assessment. Later that year, the group presented a surgical microscope-integrated HSI setup that allowed them to collect an annotated database of 52 hyperspectral images from glioma surgeries [30]. In 2023, they published corresponding results for a multitissue (blood, nodules, glioma, dura, gray matter, reflections, vein) classification where they achieved an accuracy of 80% [31].

Since the start of the Hyperspectral Imaging Cancer Detection (HELiCoiD) project in 2014, a research group at the Institute of Applied Microelectronics at the University of Las Palmas has been continuously involved in the ongoing development of intraoperative HSI imaging for intracranial procedures. During this time, they have not only collected a large amount of labeled data (over 890k labeled hyperspectral pixels) and created a repository, but they have also investigated different machine learning (ML) methods for segmenting the HSI images and classifying the hypercube pixels. In a recent paper from 2023, they presented a multiclass tissue classification method for HSI images that achieved an F1 score of 70.2 ± 7.9% for tumor tissue, normal tissue, blood vessels, and background differentiation [32]. Livermore et al. [33] as well as Jabarkheel et al. [34] analyzed ex vivo tissue using Raman spectroscopy to differentiate between normal tissue and tumor tissue, achieving sensitivities of 96% and 91.3%, as well as specificities of 99% and 81.2%, respectively. Despite the excellent detection rate, tissue already removed from the resection cavity is examined in this approach. It could provide local reassurance about the resection progress, e.g., in tumor margins, but is not equivalent to real-time in vivo visualization.

In parallel, Black et al. [35] have investigated the potential of hyperspectral fluorescence signatures as optical biomarkers for intraoperative tissue characterization (high-grade gliomas, non-glial primary tumors radiation necrosis, miscellaneous, metastases). Random forest and multilayer perceptron models achieved average test accuracies of 84–87%, 96.1%, 86%, and 91%, respectively. Beyond tissue classification, they also focused on improving the quantitative interpretation of hyperspectral fluorescence signals. Deep learning-based approaches have been proposed to correct for heterogeneous optical and geometric tissue properties and to enable more accurate estimation of PPIX concentration from hyperspectral data [36].

Similarly, Shen et al. have presented a possibility of high-throughput intraoperative assessment of ex vivo tumor specimens based on fluorescence staining using indocyanine green (ICG) in combination with deep convolutional neural network (CNN) analysis. They achieved over 90% sensitivity and over 80% specificity in differentiating tumor and non-tumor tissue [37].

Among the numerous proposed solutions to improve intraoperative vision in glioma surgery, the predominant focus is placed on improving the imaging technology. This is immensely important as it defines the basic ability to capture discriminative optical signals. Promising approaches, such as HSI in conjunction with ML can additionally offer a computational classification of the tissue in the image data but are largely still at the research stage. Recently launched microneurosurgical microscopes have primarily changed the visibility of the tumor surrounding anatomy to facilitate the procedure under blue light. Thus, a surgeon’s subjective judgment of colors and intensities is still used to evaluate vague tumor borders and ambiguous fluorescence. In this work, we aim to investigate an approach using a purely data-driven improved visualization for 5-ALA-guided resections. Herein we apply ML-based pixel classification to increase the sensitivity of the weakly and ambiguously fluorescent areas in conventional Zeiss microscope-collected RGB images. The overarching aim within this study is to explore the potential and limitations of a data-driven approach to enhance fluorescence imaging in 5-ALA-aided glioblastoma resections. This involves a dual focus:

1. Determining the sensitivity limits of neurosurgical microscope cameras in detecting PPIX fluorescence signals.
2. Establishing a method for quantifying fluorescence intensity and converting it into a clinically useful visualization.

To address these objectives, we follow a systematic approach throughout this study. We begin by utilizing controllable and reproducible synthetic PPIX samples to analyze the detectability of fluorescence signals in images acquired with conventional neurosurgical microscopes. This controlled experimental setting enables a systematic evaluation of different machine learning strategies for identifying weak fluorescence signals. Furthermore, the synthetic samples allow us to develop a model for the quantitative assessment of fluorescence intensity and its conversion into a meaningful visualization. Finally, we demonstrate the potential of the proposed approach on a small dataset of real intraoperative images, illustrating its ability to reveal subtle fluorescence patterns that may remain difficult to discern by visual inspection alone.

## 2. Materials and Methods

### 2.1. Synthetic Sample Image Acquisition

As a first step toward developing a machine learning framework for improved fluorescence detection, synthetic fluorescent samples with clinically relevant PPIX concentrations were produced and analyzed to assess the separability of fluorescence signals at the pixel level. Although the optical properties of these samples differ from those of human tissue, they provide a controlled experimental setup with known ground truth, enabling fundamental insights into the detectability of fluorescence signals in images acquired with conventional surgical microscopes. In addition, the standardized nature of this dataset allows for a systematic comparison of machine learning models and facilitates the derivation of assumptions for their subsequent adaptation to real clinical data.

**Figure 1:**
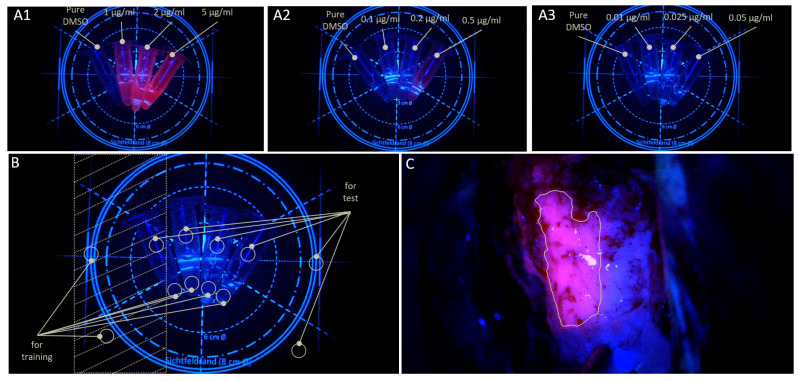
Synthetic PPIX samples under OR-microscope blue light. (**A1**–**A3**): Each contain the PPIX-free reference sample (left) and three PPIX-containing samples of different concentrations ((**A1**): 5 μg/mL, 2 μg/mL, 1 μg/mL; (**A2**): 0.5 μg/mL, 0.2 μg/mL, 0.1 μg/mL; (**A3**): 0.05 μg/mL, 0.025 μg/mL, 0.01 μg/mL); (**B**): Example of a pixel extraction (regions of interest are outlined) for the training and test set with synthetic data; (**C**): Example of a pixel extraction (region of interest is outlined) for the training set for intraoperative fluorescence detection.

Liquid fluorescent samples were prepared with nine different PPIX (Sigma Aldrich, St. Louis, MO, USA) concentrations dissolved in 1 mL dimethyl sulfoxide (DMSO; Carl Roth, Karlsruhe, Germany): 5 μg/mL, 2 μg/mL, 1 μg/mL, 0.5 μg/mL, 0.2 μg/mL, 0.1 μg/mL, 0.05 μg/mL, 0.025 μg/mL, and 0.01 μg/mL. The selected concentration range was based on values reported for malignant human gliomas following oral 5-ALA administration [38,39]. In this context, the highest concentration of 5 μg/mL represents a moderate accumulation of PPIX in grade 4 gliomas, which can be expected to produce visible fluorescence under standard surgical conditions. The remaining samples were progressively diluted to approach the detection limits of conventional fluorescence-guided surgery (FGS) imaging, reaching up to a 500-fold reduction in PPIX concentration and resulting in fluorescence levels that are no longer visually detectable. Finally, a reference sample containing pure DMSO was prepared (see Figure 1).

Sample images were acquired using the integrated camera of a microneurosurgical microscope (ZEISS KINEVO 900 S, Carl Zeiss Meditec AG, Jena, Thuringia, Germany) in fluorescence mode. For each image, three PPIX-containing samples and the reference sample were placed on a positioning template as depicted in Figure 1(A1–A3). Since the blue light illumination source of the microscope generates an uneven optical power profile within the field of view [26], the positioning template was used to obtain an approximately homogeneously illuminated area in each image. It was used to align the samples within a defined area (inner template circle) and thus ensure the same fluorescence excitation conditions in all samples.

### 2.2. Intraoperative Glioblastoma Image Acquisition

GBM images were acquired from intraoperative videos recorded during GBM fluorescence-guided resections using the microneurosurgical microscope in fluorescence mode. 5-ALA was administered to these patients 3–4 h before the start of surgery. Furthermore, images of the surgical site were collected from non-5-ALA-guided intracranial procedures, including oligodendroglioma, astrocytoma and metastasis resections. A total of 15 frames were extracted from videos of GBM resection procedures from 6 different patients and 10 frames from videos of non-fluorescence-guided procedures and 6 different patients. All procedures were performed according to standard practice using available assistive technology such as neuronavigation and ultrasound. No specifications were made for imaging system settings such as exposure intensity or microscope working distance. The aim was to reflect the preferences and variability of the surgeons with regard to individually ideal visibility in the dataset.

### 2.3. Data Preparation

The data preparation methodology was initially developed for the synthetic samples image dataset and later adapted for the intraoperative image dataset, applying uniformly to both datasets. Since the images in the datasets were provided in JPEG format, the primary focus of data preprocessing was the removal of compression artifacts, particularly block formations in dark image regions. To detect potential artifacts, a density function was approximated from the frequency distribution of pixel saturation values using a moving average filter. Saturation values whose frequency significantly exceeded the density function—by a factor greater than 1.5—were identified as likely stemming from quantization-related artifacts. Pixels exhibiting these saturation values were assigned a value of 0 in their respective color channels. These pixels were subsequently interpolated using a 9 × 9 median filter, after which the channels were reassembled into an RGB color image.

For the synthetic dataset, two non-overlapping regions of interest (ROIs) were defined in each depicted sample in all three synthetic sample images (see Figure 1B). Herein, pixels from one ROI were used for analysis and ML training, while dissimilar pixels were extracted from the second ROI for testing and evaluation purposes. This procedure yielded between 3830 and 3850 pixels (i.e., individual data points) per ROI. Additionally, two ROIs per image were defined in the dark background and two ROIs were extracted from the positioning template markers (see Figure 1B). Altogether, the synthetic dataset comprised 138,140 pixels from all ROIs, of which 69,087 pixels were allocated for training and 69,053 pixels for testing.

To construct the intraoperative dataset, regions of interest (ROIs) were manually defined in clearly fluorescent areas from five GBM images (see Figure 1C). In total, 305,132 pixels were extracted from five images obtained from three patients (Patients 1–3) and used for model training.

For the non-fluorescent (negative) class, five images acquired during non-5-ALA-guided procedures were used in their entirety without ROI specification, resulting in 10,368,000 pixels. These images were captured at different time points during the procedures of Patients 7 and 8.

Model evaluation was performed on ten frames from fluorescence-guided surgery (FGS) recordings. Five of these images originated from Patients 1–3 but were taken at different stages of the procedure, where different tissue layers were exposed compared to those used for training. The remaining five images were obtained from three additional patients (Patients 4–6).

**Figure 2:**
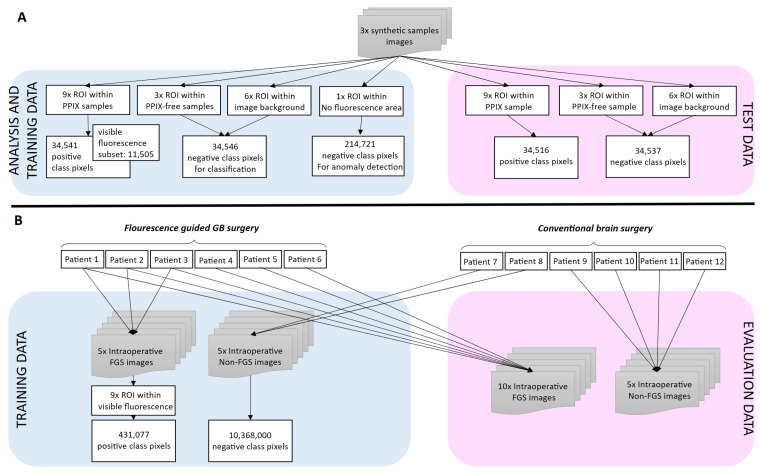
(**A**): Composition of the dataset from synthetic sample images. (**B**): Composition of the dataset from intraoperative images.

To further assess model behavior in the absence of fluorescence, five additional images from non-FGS procedures were included for evaluation, originating from Patients 9–12. A detailed overview of the composition of both datasets is provided in Figure 2.

### 2.4. Qualitative t-SNE Analysis

To provide an initial qualitative assessment of the sensitivity of FGS imaging to PPIX fluorescence and an overview of data separability, the t-distributed stochastic neighbor embedding (t-SNE) algorithm was applied to the test data, including pixels from PPIX-containing and PPIX-free samples. t-SNE projections are mostly used to visualize high-dimensional data, but they also reveal local structures in data based on similarity measures. They indicate a fundamental class separability based on non-linear relationships in the data [40]. In this study, t-SNE was utilized to evaluate class separability, offering insights into the expected performance of ML models under real-world hardware limitations. The algorithm was executed with a perplexity value of 200, using the Minkowski metric as the distance measure.

### 2.5. Model Development

To determine the sensitivity limits of neurosurgical microscope cameras for detecting PPIX fluorescence, a binary pixel-wise classification into fluorescent and non-fluorescent classes is required. However, complete annotation of intraoperative image data for training of a classification model is inherently infeasible. While clearly fluorescent areas can be annotated, vague fluorescence would require exhaustive, pixel-by-pixel manual assessments for reliable labeling—an impractical approach given the time and data volume required. Moreover, non-visible or ambiguous fluorescence cannot be annotated visually at all.

In contrast to the positive class, which is inherently challenging to annotate comprehensively, perfectly annotated negative class data—pixels depicting no fluorescence—can be generated in large quantities. This is achieved using intraoperative images acquired under blue light in neurosurgical procedures not involving glioma resection, where patients have not received 5-ALA. Against this background, we investigate, in addition to traditional classifiers, an anomaly detection approach in which the model focuses on extensively learning the negative class (or normal data), enabling it to identify abnormalities not encountered during training, specifically non-visible or ambiguous fluorescence.

Three traditional classifiers:

- Support Vector Machine (SVM) configured with a radial basis function kernel (scale = 5);
- Naïve Bayes (NB) utilizing a Gaussian probability distribution;
- Neural Network (NN) implemented as a single fully connected layer with 10 neurons and ReLU activation.

These classifiers were trained using pixels from highly fluorescent and non-fluorescent synthetic samples. In parallel, three contrastive loss Variational Autoencoder (clVAE) models with varying β-values (β = 1, 2, and 3) were trained to identify fluorescent pixels as anomalies.

#### 2.5.1. Pixel Classification

Based on the considerations outlined above, only pixels from clearly fluorescent samples were used to train the ML models—specifically from samples with PPIX concentrations of 5 μg/mL, 2 μg/mL, and 1 μg/mL. The positive class thus comprised pixels exhibiting visible fluorescence, while the negative class included pixels from the fluorescence-free reference sample as well as background pixels. PPIX-containing samples without visible fluorescence were excluded from training and were only used during testing to evaluate the model’s ability to detect weak or non-visible fluorescence signals.

To create a balanced training set, 10,000 randomly selected pixels from each class were included, resulting in a total of 20,000 data points.

#### 2.5.2. Anomaly Detection

The training set composition for the clVAE deviated from the training set of traditional classifiers. Unlike conventional classifiers, which rely on balanced training sets, the clVAE focuses on learning the negative class (non-fluorescent pixels) extensively. Thus, any amount of data from the negative class can be used without requiring balance with the positive class. For this model, 60,000 randomly selected pixels were extracted from ROIs within the non-fluorescent regions and assigned to the negative class (i.e., normal data). From this pool, 10,000 pixels were further selected at random and paired with 10,000 fluorescent pixels from the positive class (anomaly data) to create the dataset for contrastive training. Feature extraction was employed for dimensionality extension prior to training. The resulting set of 10 features, along with their respective computations, is detailed below:


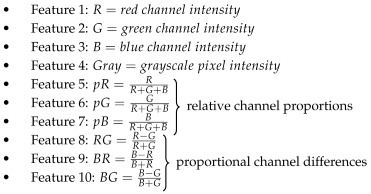


The VAE architecture comprised a 10-dimensional input layer, a three-dimensional latent layer, and a corresponding 10-dimensional output layer. Training was conducted over 1000 epochs, with calculations performed on randomly selected mini-batches of size 256 within each epoch. The learning rate was progressively reduced from 0.001 to 0.0001 following an exponential decay schedule. The β-factor, which controls the balance between reconstruction accuracy and latent space regularization, was adjusted using a two-phase cyclic annealing algorithm spanning a total of 20 cycles. During the first half of each cycle, β increased from 0 to its maximum value following a sigmoidal progression. In the second half of each cycle, β remained constant at this maximum value. Three distinct VAE models were trained for anomaly detection, each with a different maximum β-factor, including β = 1, β = 2, and β = 3. The Kullback–Leibler divergence was used as the anomaly score for normal and anomaly classification.

#### 2.5.3. PPIX Quantification

In addition to detecting fluorescence, an objective quantification of signal intensity is essential for assessing the biological relevance and potential malignancy of tissue. To this end, a predictive model was developed to estimate the underlying PPIX concentration of each pixel, using the synthetic samples as a calibrated reference system with known fluorophore content. All training pixels from PPIX-containing samples, represented with their extended feature set (see Section 2.5.2), were included to develop a robust quantification model for predicting the PPIX concentration corresponding to each pixel. To begin, the predictive power of individual features for concentration estimation was assessed using a random forest (RF) as an evaluation tool.

The fit quality of the RF was determined through out-of-bag (OOB) prediction, which provides a reliable measure of model performance without requiring a separate validation set. Feature importance scores were derived based on the OOB predictor importance estimates using permutation, highlighting the most influential features for PPIX concentration prediction. Subsequently, the strongest predictors identified by the RF were used to obtain a PPIX concentration prediction model using polynomial fitting.

**Figure 3:**
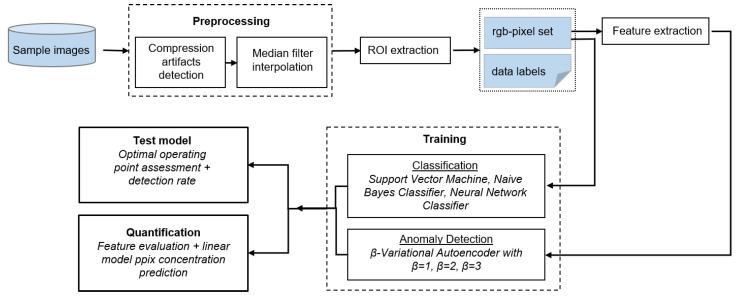
Data processing and model development workflow.

Figure 3 visualizes the complete ML-model and quantification model development workflow including data preparation.

### 2.6. Model Transfer and Evaluation

Following the model comparison presented above, the β = 1 clVAE was selected for application to real intraoperative data. In this new training phase, 500,000 randomly selected pixels extracted from non-FGS surgery images were used as normal data, accompanied by 50,000 normal–anomaly pairs (see Section 2.3 for details on the intraoperative dataset). The remaining hyperparameters were carried over from the training process designed for the labeled PPIX-sample dataset.

**Figure 4:**
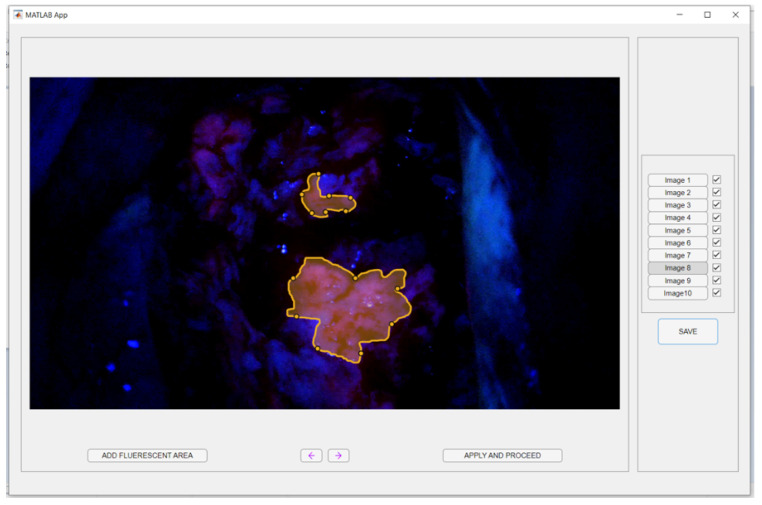
Custom annotation application GUI. Two exemplary annotated regions are outlined (orange borders).

The model’s false positive rate was initially determined by evaluating pixels classified as anomalies within the five test set images from non-FGS interventions. Additionally, four experienced surgeons were invited to annotate regions in a selection of 10 images from FGS procedures wherever they could still visually discern fluorescence. The regions they annotated were compared with the areas identified as anomalies by the clVAE model to assess the model’s ability to detect subtle or non-visible fluorescence and to explore its potential for providing a more sensitive and objective tool for real-time surgical guidance in fluorescence-aided GBM resections. To facilitate this, a custom application was developed using the Matlab App Designer Tool (version R2024a, MathWorks, Natick, MA, USA), providing the surgeons with user-friendly annotation capabilities and saving annotations as logical masks. A screenshot of its graphical user interface (GUI) is shown in Figure 4.

Surgeons were instructed to adjust their monitor brightness to optimize the visibility of fluorescence in the test images. The regions they annotated were compared with the areas identified as anomalies by the clVAE model to assess the model’s ability to detect subtle or non-visible fluorescence and to explore its potential for providing a more sensitive and objective tool for real-time surgical guidance in fluorescence-aided GBM resections.

## 3. Results

### 3.1. Qualitative Assessment of Fluorescence Detectability in Synthetic Samples

**Figure 5:**
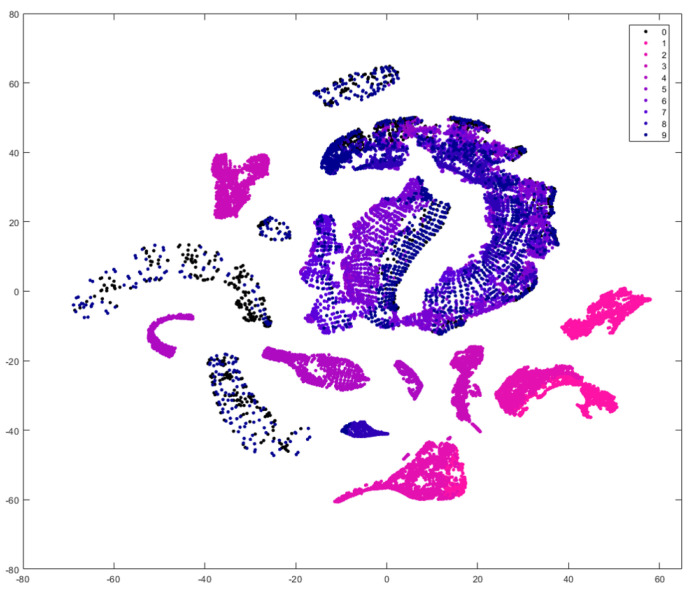
t-SNE projection of the synthetic sample data. PPIX concentration are color-coded from pinkt to blue shades. PPIX-free reference sample data is depicted as black dots.

Figure 5 shows the t-SNE projection of synthetic sample pixels providing a qualitative analysis of general identifiability of fluorescence signals based on the pixel information content. Data pixels from PPIX-containing samples are color-coded in pink and blue according to their respective PPIX concentrations, while pixels from PPIX-free samples are represented by black dots. A clear separability of high-dose, visibly fluorescent samples is evident, with distinct clusters emerging for these concentrations. Similarly, vaguely fluorescent samples with 0.5 μg/mL PPIX form clearly differentiated clusters, with minimal overlap with the reference (PPIX-free) sample. Lower concentrations of PPIX, which do not produce visible fluorescence under the operating microscope, also exhibit partial differentiation from the reference sample. Separate distributions are observed at 0.2 μg/mL and 0.1 μg/mL, although these show some contamination with reference sample data points. The lowest concentration of 0.01 μg/mL, however, is indistinguishable from the reference sample based solely on pixel information, indicating that the fluorescence signal at this level is too weak for differentiation by a pixel data-driven model.

### 3.2. Classification and Anomaly Detection Models Yield Fluorescence Detection Below Visual Perception Threshold in Synthetic Fluorescent Samples

**Figure 6:**
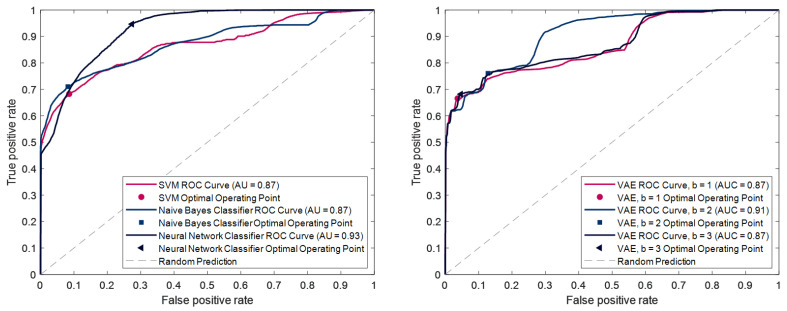
ROC curve for the tested classifier models (left hand side) and the anomaly detection models (right hand side).

Figure 6 summarizes the performance of the investigated ML models on the synthetic test set—comparing all PPIX concentrations against the reference sample and image background—using Receiver Operating Characteristics (ROC) curves. The consistently high classification results indicate that the models effectively learn to discriminate between fluorescent and non-fluorescent pixels, even without prior exposure to vague or non-visible fluorescence data. This demonstrates the potential of ML-based methods for detecting fluorescent regions in image data. All classifiers achieve comparably high Area under the Curve (AUC) values, ranging from 0.87 to 0.93, with the neural network emerging as the best-performing classifier. Table 1 provides a detailed breakdown of the results. It includes the AUC, Accuracy, Sensitivity, and Specificity for each model at the optimal operating point of the ROC curves. The Neural Network Classifier achieves the highest AUC and Accuracy scores. However, its high sensitivity is offset by a relatively lower specificity of 0.73.

For a more purposeful comparison of the models, the second part of the table demonstrates the percentage detection rate of pixels according to the underlying PPIX concentration. For this purpose, the classification and detection thresholds of all models were set to a conservative level, which corresponds to a specificity of 99%. These results show that all models can reliably detect both visible and vague fluorescence. However, significant differences emerge in the detection of non-visible fluorescence, where the anomaly detection approach (clVAE) demonstrates a clear advantage. At a PPIX concentration of 0.2 μg/mL, the clVAE models detect over 20 percentage points more pixels than any traditional classifier. At 0.1 μg/mL, they surpass SVM and NN by a similar margin and achieve slightly better results than NB.

The best-performing clVAE model (β = 1) achieves remarkable results: it detects over two-thirds of pixels with 0.2 μg/mL PPIX and nearly half of the pixels at 0.1 μg/mL—both not emitting visible fluorescence. Even at a 100-fold dilution of the initial sample (0.05 μg/mL), the model correctly identifies isolated pixels, achieving a detection rate of 3.6%.

### 3.3. Development of a PPIX-Fluorescence Quantification Model

**Figure 7:**
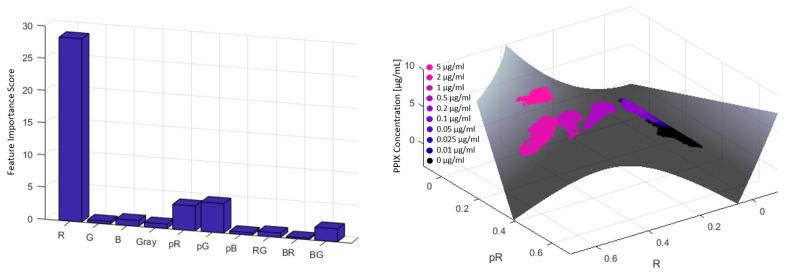
(**Left**): Feature Importance Scores derives from Random Forest fitting for the extended feature set. (**Right**): Polynomial model fitted plane along with pixel data distribution. The underlying PPIX concentration of individual data points is color-coded in pink, blue and black colors.

The RF model used for feature evaluation achieved a $R^{2}=0.99$, indicating a nearly perfect fit. The bar plot in Figure 7 (left-hand side) presents the feature importance analysis derived from the RF model, revealing that the PPIX concentration is predominantly associated with the red channel intensity (R). This finding aligns with the design specifications of neurosurgical microscopes, which employ optical filters specifically tuned to the red emission spectrum of PPIX, thereby amplifying signal intensity in the red channel during fluorescence-guided surgery.

A quadratic model, using only the red channel intensity as a predictor, was constructed:

$$f\left(x\right)=p1+p2*x+p3*x^{2}$$

with the following coefficients and 95% confidence bounds:

- p1 = 2.166 (1.742, 2.591);
- p2 = 7.325 (7.166, 7.484);
- p3 = −0.02339 (−0.02895, −0.01784).

This model achieved a limited fit with R^2^ = 0.72, indicating that the red channel alone explains only part of the variance in PPIX concentration.

To improve the model, the second most relevant feature identified by the RF analysis—the proportional green channel component (pG)—was added as a second predictor. The resulting polynomial model is described by

$$f\left(x,y\right)=p00+p10*x+p01*y+p20*x^{2}+p11*x*y$$

where the coefficients and their 95% confidence bounds are as follows:

- p00 = 0.0327 (0.02887, 0.03653);
- p10 = −1.009 (−1.104, −0.9135);
- p01 = 0.6343 (0.6005, 0.6682);
- p20 = 46.99 (46.67, 47.31);
- p11 = −93.46 (−93.92, −93).

By incorporating both features R and pG, the model’s fit significantly improved, achieving $R^{2}=0.92$. This indicates a robust relationship between these two features and the PPIX concentration in the corresponding pixel, and the model effectively explains the variability in the data. Figure 7 (right-hand side) illustrates the distribution of pixel data in the R-pG feature space in a three-dimensional scatter plot, along with the fitted plane from the polynomial model. The visualization underscores the clear relationship between these features and PPIX concentration, highlighting the potential of this approach for accurate quantification of fluorescence intensity.

### 3.4. Fluorescence Identification Capacity of Anomaly Detection Model Transferred to Intraoperative Images

**Figure 8:**
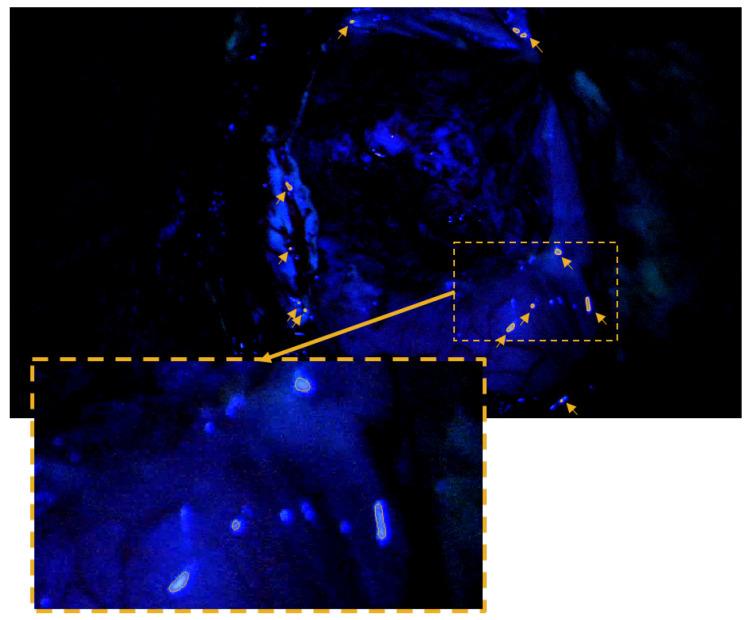
Example of non-FGS image with clVAE-detected false positive fluorescent areas (outlined by orange line and indicated by small orange arrows). Image corresponds to image No. 2 (Patient 10) in Table 2.

One of the test images from non-fluorescence-guided, conventional intracranial procedures is shown as an example in Figure 8. Areas falsely recognized as fluorescence by the VAE model are outlined and their locations are additionally marked with arrow symbols. The false positives in the test image are characterized by small areas that occur in well-illuminated areas of the image. In the zoomed-in representation, it can be seen that those areas appear on light reflections that occur on the tissue surface. A quantitative summary of the false positive pixels detected across all five test images is provided in Table 2. The absolute number of false positive pixels is consistently a small fraction of the total 2,073,600 pixels in each image. Column 4 of the table presents the specificity of the model for each individual image. The specificity is calculated based on all image pixels, with an additional evaluation excluding the black pixels (where the color channels are red = 0, green = 0, and blue = 0) in parentheses. This provides a more conservative and meaningful assessment of model performance, particularly given that large portions of the image are dark and contain non-informative black pixels. Regardless of the calculation method, the model demonstrated impressive performance, correctly classifying over 99.9% of all pixels in the test images, yielding an overall specificity of 99.96%.

**Figure 9:**
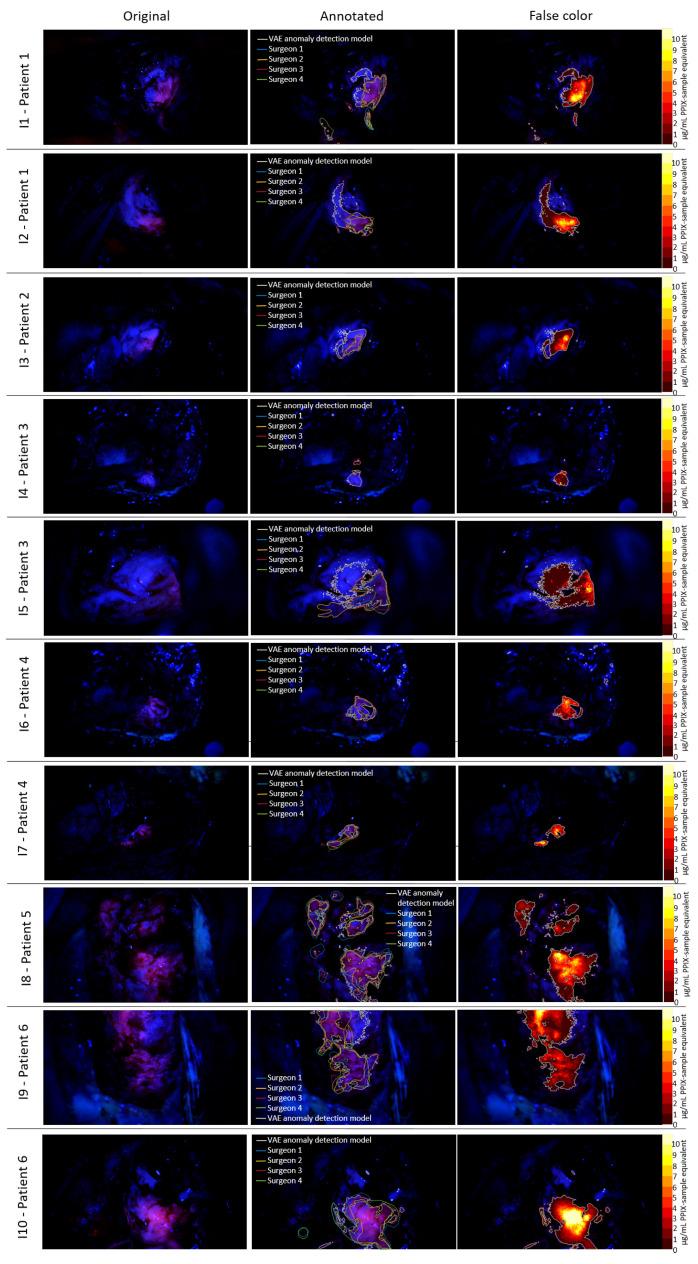
FGS test images. (**Left column**): original FGS microscope image; (**middle column**): surgeon annotations of fluorescence along with clVAE detected fluorescence; (**right column**): overlay of the image with clVAE-detected fluorescence and color-mapped fluorescence intensity.

Figure 9 shows the results of pixel-by-pixel classification using the clVAE with β = 1 model on 10 intraoperative images from FGS resections. The first column displays the original images, the second column shows the images with outlines indicating the fluorescent areas recognized by the model and the surgeons’ annotated fluorescence. The third column illustrates the fluorescence quantification using the method outlined above, based on the synthetic samples. The functional capability of the model is demonstrated by the generally high overlap between the areas annotated as visibly fluorescent by the surgeons and those identified by the model. The model successfully detects all visibly fluorescent regions, and in some cases, it identifies larger areas of fluorescence, including regions with vague or visually imperceptible fluorescence that were not annotated by the surgeons. This is particularly evident in Figure 9 I1–I5 and I9, where the model’s identified areas are up to 2.4 times larger than the annotations made by the surgeons. The fluorescence quantification approach shown in the third column aligns well with visual observations. Clearly visible fluorescence corresponds to PPIX-sample equivalents of 5 μg/mL, while the vaguely fluorescent regions are associated with lower concentrations. Areas that do not exhibit visible fluorescence and were not identified as such by the surgeons are quantified with values ranging from 0 to 2 μg/mL PPIX-sample equivalent. Particularly in Figure 9 I6, smaller areas detected on reflective tissue in the upper left corner of the image are observed. As this tissue lies outside the actual resection cavity, on the intact dura surface, these regions are likely false positives. However, the frequency and pattern of occurrence of these false positives align with those previously observed in the specificity considerations, indicating that the model’s specificity remains consistent across different cases.

## 4. Discussion

The relationship between tumor cell burden and disease progression, coupled with the observation that residual glioma cells remain at the resection margins in approximately half of FGS cases, underscores the need for improved intraoperative imaging techniques capable of reliably delineating high-grade gliomas.

The new generation of microneurosurgical imaging systems has already introduced advancements in fluorescence modules. Leica Microsystems, for example, has introduced two new visualization modes within its GLOW400 AR application for 5-ALA-FGS, both based on multispectral imaging [41]. The Anatomy view mode provides a clear vision of the surrounding anatomy and eliminates the need to switch between white light and blue light. The Highlighted Fluorescence View mode is designed to enhance the visibility of weak fluorescence against the background, although formal evaluation of its effectiveness is currently lacking. The Olympus Orbeye Exoscope from Olympus similarly enhances anatomical visibility through image processing, even under poor lighting conditions. For detecting fluorescent tumor areas, the Orbeye system demonstrated a sensitivity of 75% and a specificity of 80% [42]. Suero Molina et al. [16] have presented a further development of the 5-ALA fluorescence module in Zeiss surgical microscopes named BLUE 400 AR mode. Here, enhanced visibility in FGS mode is achieved through an improved filter system and fluorescence detection was found to have a sensitivity of 70.89% and a specificity of 97.37%.

While current and ongoing efforts focus on enhancing microneurosurgical imaging devices with advanced camera and image acquisition technologies, we propose augmenting existing systems with an ML-based approach for pixel-wise image assessment and real-time visualization.

In this study, we demonstrated the extensive potential of a VAE-based anomaly detection approach for more sensitive and objective fluorescence detection and visual indication. Experimental investigations incorporating synthetic fluorescent samples of known ground truth revealed that ML models could learn the correct patterns to identify weak or non-visible PPIX fluorescence in FGS imaging pixels, even when trained solely on data containing strong fluorescence signals. At a PPIX concentration of 0.1 μg/mL represented in a pixel, fluorescence was detected in about half of the test data samples—a concentration that is five times lower than the concentration invoking weak, faintly visible fluorescence.

Further, the fluorescent regions identified by the clVAE extended well beyond the surgeon-annotated areas by visual assessment—often by a significant margin. Although pixel-wise ground truth was unavailable for the images used in this study, it is highly plausible that the areas detected by the clVAE are based on a learned fluorescence fingerprint. Beyond the experimental data, this is further supported by the expansion of the detected areas, which not only encompass the surgeon-annotated regions but also the fading fluorescence at their periphery, extending into well-illuminated tissue. Importantly, the detected areas differed markedly from false positives found in the images from non-fluorescence-guided procedures. The latter were typically small, scattered, and primarily associated with reflections on the tissue surface.

However, it is important to note that this paper presents a set of preliminary results and serves as a proof of concept. Consequently, the study has certain limitations, particularly due to the restricted dataset used. Another notable limitation is the model’s reliance solely on pixel-specific features as input data. Future iterations of this approach should incorporate a more extensive dataset to improve sensitivity by including additional feature types. Specifically, integrating local features from neighboring pixels and global features that capture contextual information from the entire image could provide valuable insights for ML models. This extended contextual data has the potential to significantly enhance model performance and reliability.

The evaluation of the clVAE model’s specificity yielded an excellent value exceeding 99.9%. However, the limited amount of test data poses a significant constraint on the validity of these results. The accumulation of false positives associated with light reflections on tissue surfaces is a plausible finding. False positives were primarily associated with specular reflections on wet tissue surfaces. Such reflections can produce high signal intensities and partial sensor saturation across color channels, particularly with strong red components that resemble fluorescence signals. Addressing these artifacts will be essential for clinical applicability. Approaches such as cost-sensitive learning or hard-negative mining may help the model better distinguish true fluorescence signals from reflection-induced artifacts.

The quantification model presented in this study, aimed at modeling fluorescence intensity and providing a corresponding visualization, demonstrates promising qualitative results. Typically, a high-intensity nucleus is observed, corresponding to the visible fluorescence in the original images, with a gradual decrease in intensity toward surrounding tissue. This pattern is consistent with the infiltrative growth behavior characteristic of high-grade glioma. The assumption that fluorescence intensity correlates with tumor cell density—and more specifically with the concentration of PPIX within the tissue volume—is intuitive. However, several factors, including exposure intensity, shadowing effects, the time elapsed since 5-ALA administration, and interpatient variability, cannot be inferred from pixel-level information alone. Consequently, the conclusions drawn from the model remain qualitative in nature.

Importantly, the displayed fluorescence intensity cannot be considered a direct surrogate for tumor cell density and should therefore not be interpreted as a recommendation for further tissue resection. Particularly in eloquent brain regions, surgical decision-making must remain guided by established functional and anatomical constraints, with ML-based fluorescence enhancement serving only as an additional source of information to support intraoperative assessment.

Despite these limitations, objective, data-driven classification methods hold significant potential for supporting intraoperative decision-making. This is particularly relevant in critical, eloquent brain areas, where cytoreduction requires precise and informed considerations to balance tumor removal with the preservation of healthy tissue.

Comparable clinical challenges exist in other oncologic disciplines where complete tumor resection or accurate tissue identification is crucial for patient prognosis. In gynecologic oncology, fluorescence-guided surgery is routinely used for sentinel lymph node mapping in endometrial, cervical, and vulvar cancer, typically employing indocyanine green (ICG). Similar applications are found in thoracic surgery for non-small cell lung cancer using near-infrared fluorescence imaging. While artificial intelligence is already widely applied in these fields for diagnostic imaging, radiomics-based staging, lymph node classification, and treatment planning, the direct AI-assisted interpretation of intraoperative fluorescence imaging remains limited. Current intraoperative applications are often constrained by challenges such as limited availability of well-annotated datasets and regulatory requirements for clinical decision-support systems [43].

Nevertheless, recent studies have explored advanced optical imaging techniques combined with machine learning to address these limitations. In addition to hyperspectral imaging approaches that have been investigated particularly in neurosurgery, other modalities such as fluorescence lifetime imaging combined with ML models like SVM, random forests, and CNNs have shown promise for tumor detection in oral and oropharyngeal cancer [44]. Likewise, AI-assisted analysis of ICG perfusion videos has demonstrated encouraging results in rectal cancer for the demarcation of tumor and healthy tissue [45]. Beyond intraoperative imaging alone, multimodal AI approaches integrating radiological imaging, pathological features, and molecular or omics data are increasingly investigated to provide a more comprehensive representation of disease characteristics and may enable applications such as real-time pathological grading of gliomas [37].

These developments highlight both the potential and the current limitations of machine learning in surgical decision support. While AI can enhance image interpretation, improve tissue classification, and integrate complex multimodal information, its clinical deployment remains limited by data availability and the need for robust validation. In this context, the presented approach aims to contribute to the emerging field of AI-assisted fluorescence-guided surgery by enabling sensitive fluorescence detection using standard RGB microscope images without requiring specialized imaging hardware.

## 5. Conclusions and Future Perspectives

This study demonstrates the feasibility of using machine-learning-based anomaly detection to enhance the visualization of PPIX fluorescence in conventional RGB images acquired with surgical microscopes. Even with limited training data the proposed approach was able to detect weak fluorescence signals beyond the limits of visual perception and highlight extended regions potentially associated with infiltrative tumor tissue.

We propose this system as a lightweight software plugin for real-time fluorescence evaluation, designed for integration into existing operating room workflows. This approach has the advantage of requiring no major hardware modifications and could therefore enable faster and more cost-effective clinical adoption compared with emerging hardware-based solutions such as multispectral imaging systems.

In future work, larger and more diverse intraoperative datasets will be essential to further validate and refine the proposed models. Incorporating contextual image features, improving robustness against reflection artifacts, and systematically evaluating model performance across different surgical conditions will be important steps toward clinical translation. In addition, direct comparisons with spectroscopic imaging techniques should be performed to determine whether ML-based analysis of conventional RGB data can achieve comparable diagnostic performance.

Ultimately, integrating real-time, ML-assisted decision support into surgical microscopes could enhance intraoperative safety, support more complete tumor resections, improve workflow efficiency, and, eventually, contribute to better patient outcomes.

## Figures and Tables

**Table 1:** Model assessment at optimal ROC operating point as well as at a fixed specificity of 99%.

	Classification at				Detection Rate at Different Concentrations [μg/mL]								
	**the Optimal Operating Point**				**and 99% Specificity**								
	**AUC**	**Acc.**	**Sens.**	**Spec.**	**5**	**2**	**1**	**0.5**	**0.2**	**0.1**	**0.05**	**0.025**	**0.01**
SVM	0.8653	0.7985	0.6826	0.9142	100	100	100	100	43.52	21.29	5.37	1.35	0.00
NB	0.8716	0.8143	0.7103	0.9183	100	100	100	100	46.61	41.61	1.09	0.00	5.58
NN	0.9268	0.8365	0.9469	0.7261	100	100	100	100	13.68	5.21	5.79	0.68	0.00
β = 1 VAE	0.8658	0.8135	0.6656	0.9644	100	100	100	100	67.78	48.20	3.60	0.57	0.65
β = 2 VAE	0.9098	0.8163	0.7604	0.8721	100	100	100	100	68.84	43.59	3.75	0.68	0.05
β = 3 VAE	0.8719	0.8177	0.6818	0.9536	100	100	100	100	66.84	43.54	3.81	0.89	0.05

**Table 2:** False positive detected pixels per non-FGS test image and corresponding specificities.

Image	Patient	FP	Specificity
**No.**	**No.**	**Pixel**	**(Excluding Black)**
1	9	915	0.9996 (0.9995)
2	10	1209	0.9994 (0.9991)
3	11	215	0.9999 (0.9994)
4	12	472	0.9998 (0.9997)
5	12	907	0.9996 (0.9993)

## Data Availability

The image data generated and analyzed during the current study are openly available in the Zenodo repository at https://doi.org/10.5281/zenodo.15260349 (accessed on 25 March 2026). The code used for model development in this study is openly accessible on GitHub at https://github.com/AnnaSchaufler/Fluorescence-Assessment-in-FGS (accessed on 25 March 2026).
